# Oral health-related quality of life in patients with stroke: a randomized clinical trial of oral hygiene care during outpatient rehabilitation

**DOI:** 10.1038/s41598-017-07666-y

**Published:** 2017-08-09

**Authors:** Ruoxi Dai, Otto L. T. Lam, Edward C. M. Lo, Leonard S. W. Li, Colman McGrath

**Affiliations:** 1 Department of Dental Public Health Faculty of Dentistry, The University of Hong Kong, 3/F Prince Philip Hospital, 34 Hospital Road, Sai Ying Pun, Hong Kong; 2 The Second People’s Hospital of Hefei, 246 Heping Road, Hefei, Anhui China; 3 Department of Prosthodontics, Faculty of Dentistry, The University of Hong Kong, 4/F Prince Philip Hospital, 34 Hospital Road, Sai Ying Pun, Hong Kong; 4 Department of Rehabilitation Medicine, Tung Wah Hospital, 12 Po Yan Street, Sheung Wan, Hong Kong China

## Abstract

This study was to evaluate the effectiveness of oral hygiene care in improving oral health- and health-related quality of life (OHRQoL and HRQoL) among patients receiving outpatient stroke rehabilitation. Subjects were randomized to: (1) a conventional oral hygiene care programme (COHCP) comprising a manual toothbrush, and oral hygiene instruction, or (2) an advanced oral hygiene care programme (AOHCP) comprising a powered toothbrush, 0.2% chlorhexidine mouthrinse, and oral hygiene instruction. The interventional period lasted for 3 months, followed by a 3-month observational period. HRQoL was assessed by SF-12, and OHRQoL was assessed by Oral Health Impact Profile-14 (OHIP-14), General Oral Health Assessment Index (GOHAI), and Oral Health Transitional Scale (OHTS). Participants in AOHCP group had significantly better OHRQoL at the end of clinical trial as assessed by OHTS (p < 0.01), and at the end of observational study as assessed by GOHAI (p < 0.05) than those in the COHCP. Participants in the AOHCP group had significantly better HRQoL as assessed by physical component summary score (PCS) the end of both 3 and 6 months (both p < 0.05). This study provided the evidence that the AOHCP was more effective than the COHCP within stroke rehabilitation in improving subjective health.

## Introduction

Stroke is the predominant cause of permanent disability among the elderly^1^. Post-stroke impairments (motor, perceptual, and cognitive) can have negative impacts on daily functions, and thus affect health-related quality of life (HRQoL)^2, 3^. Likewise, oral health-related quality of life (OHRQoL) is also affected considerably as a result of post-stroke orofacial impairments^4^. Reduced tongue pressure fails to propel the food bolus into the pharynx. Decreased lip force causes drooling during mastication. Hyposalivation results in a lack of lubrication to the oral mucosa. Abnormal oral sensory function causes choking and aspiration as result of inaccurate estimation of the food bolus size. Discordant orofacial function leads to low chewing efficiency, limited food choice, and malnutrition. Embarrassment, low self-esteem, and discomfort may also arise.

12-item Short Form Health Survey (SF-12) has been used widely as a generic HRQoL measure among patients with stroke due to the fewer items and associated reduced burden on subjects^5^. The Oral Health Impact Profile-14 (OHIP-14)^6^ and the General Oral Health Assessment Index (GOHAI)^7^ are among the most commonly used measures to investigate OHRQoL among patients with stroke. The GOHAI is better at detecting functional aspects, while the OHIP-14 is better at identifying psychosocial problems^8^. The oral health transition scale (OHTS) is another OHQoL instrument that gauges the patient’s perceptions before and after the stroke regarding several aspects of oral health^9^. Together they offer complementary information on OHRQoL and changes among patients with stroke.

A number of clinical trials on oral hygiene care programmes (OHCP) among patients with stroke have been reported over the past decade^10–14^; however, only one trial reported HRQoL and OHRQoL as one of the outcomes to evaluate the effectiveness of OHCP among hospitalized patients with stroke^13^. Unlike those objectively measured clinical outcomes, the assessment of HRQoL and OHRQoL provide insight from patients’ own perspectives of how OHCP impacts on their health status, and thus is crucial in evaluating the effectiveness of OHCP. To this end, this study aims to determine and compare the effectiveness of two OHCP interventions in improving HRQoL and OHRQoL in patients following stroke during outpatient rehabilitation.

## Results

### The profile of subjects

**Figure 1:**
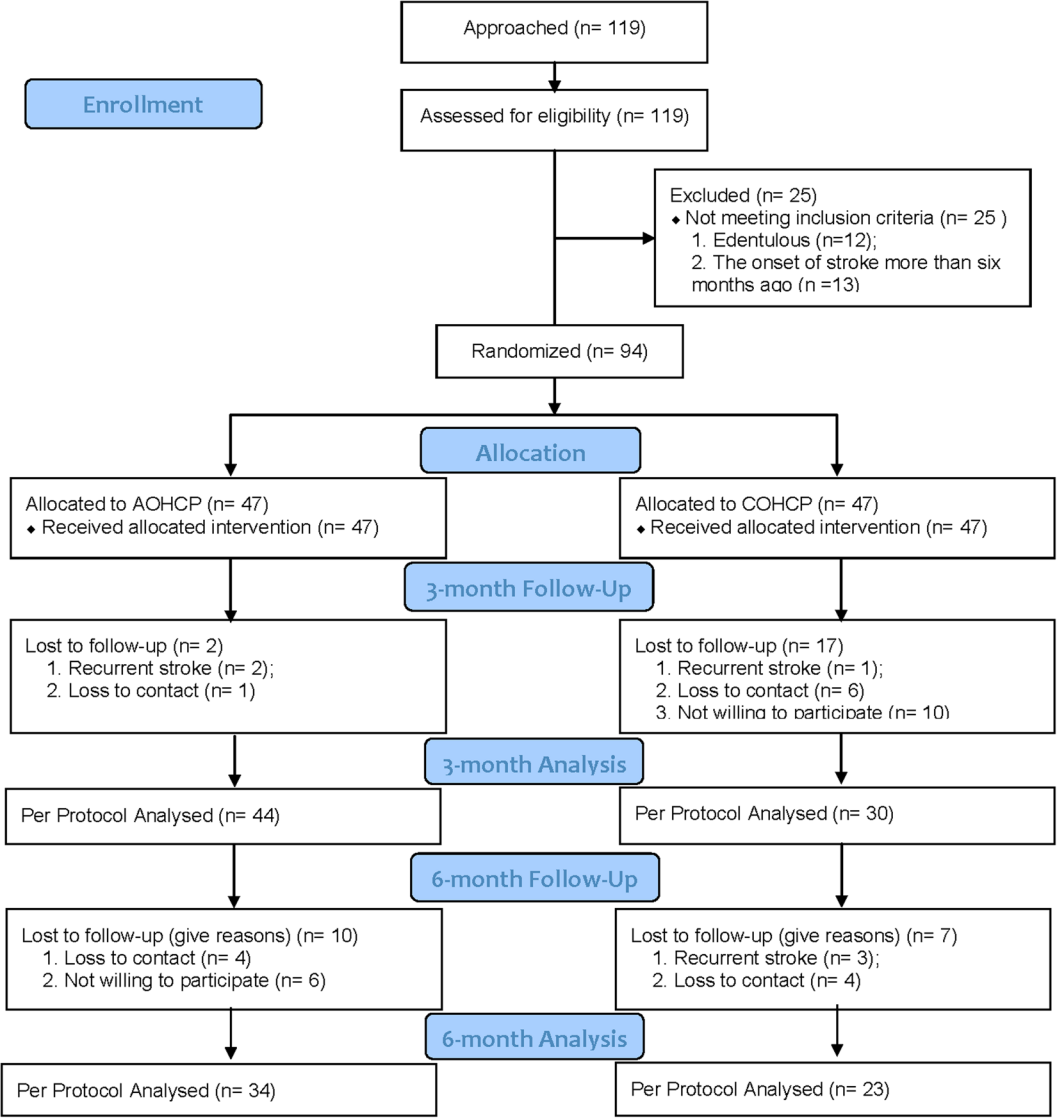
Flow diagram of numbers of patients undergoing stroke rehabilitation in different stages of the study.

A total of 94 patients were recruited at baseline, 78.7% (74/94) of subjects were assessed at 3 months, and 60.6% (57/94) of subjects were assessed 3 months after the clinical trial. The reasons for loss to follow-up included hospitalization for recurrent stroke, loss of contact with the centre (death/ moved away/ other reasons), and unwilling to participate. The numbers of subjects in different stages of the study was presented in Fig. 1 according to the statement of *Consolidated Standards of Reporting Trials 2010 (CONSORT 2010)*. Information regarding *CONSORT 2010 checklist* was presented in *Appendix I*.

There were no significant differences in the profiles of demographics and objectively-measured oral health status (Decay, Missing, Filled Tooth (DMFT), Community Periodontal Index (CPI), Loss of Attachment (LOA), oral mucosa, and denture wearing) between the AOHCP and COHCP group at baseline (p > 0.05). Those who remained in the study at the end of 3 months were similar to those who were lost to follow-up in the profiles of demographics and objectively-measured clinical oral health status (p > 0.05).

### Oral health-related quality of life

Among all subjects the median GOHAI score was 54.0 at baseline (IQR: 49.0, 56.0) and 55.0 (interquartile range (IQR): 50.8, 58.0) at 3-month review. There was a significant increase in GOHAI scores over 3 months within the AOHCP group (p < 0.05) while there was no significant change in GOHAI scores over time among those who received COHCP (p > 0.05). Regarding OHIP-14, among all subjects the median OHIP-14 score was 4.0 (IQR: 2.0, 12.0) at baseline and 3.5 (IQR: 1.0, 8.3) at 3-month assessment. There was a significant decrease in OHIP-14 scores over 3 months among those who received AOHCP (p < 0.01) while there was no significant change in OHIP-14 scores over time among those who received COHCP (p > 0.05). Regarding OHTS, among all subjects the median OHTS score was 3.0 (IQR: 1.0, 5.0) at baseline and 2.0 (IQR: 1.0, 3.0) at 3 months. There was a significant decrease in OHTS scores over 3 months among those who received AOHCP (p < 0.001) while there was no significant change in OHTS scores over time among those who received COHCP (p > 0.05). There was no significant difference in OHRQoL scores between the two groups for GOHAI, OHIP-14, and OHTS scores both at baseline and 3-month assessment (p > 0.05).

Regression analyses findings of factors associated with GOHAI scores is presented in Table 1. The intervention was not associated with GOHAI scores at 3 months (p > 0.05); however, it was associated with GOHAI scores at 6 months (p < 0.05). For those who received the AOHCP their GOHAI scores at 6 months was higher than those who received the COHCP by a value of 1.05. The other factors significantly associated with GOHAI scores at both 3 and 6 months were baseline GOHAI score, and the percentage of sites with moderate to abundant plaque at baseline. For every increase in GOHAI scores at baseline by a value of 1, GOHAI score at 3 months was increased by a value of 0.65 (p < 0.001). Every increase in the percentage of sites with moderate to abundant plaque at baseline was associated with a decrease in GOHAI score at 3 months by a value of 0.06 (p < 0.01). Similar effects of the two factors on GOHAI at 6 months score were also observed.

**Table 1:** Factors associated with GOHAI (linear regression). 3-month final model: adjusted R square = 0.64. 6-month final model: adjusted R square (model 3) = 0.57.

Model	Variable	Unstandardized coefficient	SE	Standardized coefficient	p-value	Collinearity statistics	
						Tolerance	VIF
**3 months**	Intervention	−1.05	0.68	−0.1	0.13	1	1
	GOHAI baseline	0.65	0.06	0.74	<0.001	0.97	1.03
	Plaque baseline	−0.06	1.8	−0.2	<0.01	0.97	1.03
**6 months**	Intervention	−1.91	0.8	−0.16	0.019	1	1
	GOHAI baseline	0.67	0.07	0.7	<0.001	0.97	1.03
	Plaque baseline	−0.05	0.02	−0.17	0.016	0.97	1.03

Regression analyses findings of factors associated with OHIP-14 scores is presented in Table 2. The intervention was not associated with OHIP-14 scores at both 3 and 6 months (p > 0.05). Baseline OHIP-14 score was significantly associated with OHIP-14 scores at 3 months. For every increase in baseline OHIP-14 score by a value of 1, the OHIP-14 score was 1.11 times (IRR: 1.11) of the original score (p < 0.001). Similar effect of the baseline OHIP-14 score on OHIP-14 score at 6 months was also observed.

**Table 2:** Factors associated with OHIP-14 (negative binomial regression). 3-month final model: Omnibus Test: p < 0.001; Goodness of fit: deviance/df = 0.75; Pearson Chi-square/df = 0.66. 6-month final model: Omnibus Test: p = 0.003; Goodness of fit: deviance/df = 1.14; Pearson Chi-square/df = 0.73.

Model	Variable	IRR	SE	p-value
**3 months**	Intervention	1.13	0.18	0.47
	OHIP-14 baseline	1.11	0.01	<0.001
**6 months**	Intervention	1.26	0.23	0.19
	OHIP-14 baseline	1.1	0.37	<0.001

Regression analyses findings of factors associated with OHTS scores is presented in Table 3. The intervention was significantly associated OHTS scores at 3 months but not at 6 months. The OHTS scores was 0.82 less in those who were in the group of AOHCP compared to those who were in the group of COHCP (p < 0.01). The other factors significantly associated with OHTS scores both at 3 and 6 months were number of comorbidities, and baseline OHTS score. Every increase in numbers of comorbidities by a value of 1 was associated with an increase in OHTS scores at 3 months by a value of 0.43 (p < 0.05). Every increase in OHTS scores at baseline by a value of 1 was associated with an increase in OHTS scores at 3 months by a value of 0.66 (p < 0.001). Similar effects of the aforementioned two factors on OHTS at 6 months were also observed. Wearing a denture at baseline was associated with an increase in OHTS scores at 3 months by a value of 0.83 (p < 0.05).

**Table 3:** Factors associated with OHTS (linear regression).

Model	Variable	Unstandardized coefficient	SE	Standardized coefficient	p-value	Collinearity statistics	
						Tolerance	VIF
**3 months**	Intervention	0.82	0.30	0.19	0.007<	0.97	1.04
	OHTS baseline	0.66	0.06	0.71	<0.001	0.98	1.03
	Comorbidities	0.43	0.16	0.18	0.010	0.97	1.03
	Denture	0.83	0.36	0.16	0.024	0.99	1.01
**6 months**	Intervention	0.64	0.36	0.14	0.08	0.97	1.03
	OHTS baseline	0.60	0.08	0.63	<0.001	0.98	1.03
	Comorbidities	0.40	0.19	0.17	0.04	0.97	1.03

3-month model: adjusted R square = 0.64. 6-month model: adjusted R square = 0.64.

### Health related-quality of life

Regarding HRQoL scores, among all subjects the median physical component summary score (PCS) was 33.3 (IQR: 27.8, 38.9) at baseline and 39.1 (IQR: 32.8, 48.0) at 3-month assessment. There was a significant increase in PCS scores over 3 months within each group (AOHCP: p < 0.001, COHCP: p < 0.05). Among all subjects the median mental component summary score (MCS) was 43.7 (IQR 37.1, 55.2) at baseline, and 49.7 (IQR 41.4, 55.4) at 3-month assessment. There was a significant increase in MCS scores over 3 months within each group (AOHCP: p < 0.05, COHCP: p < 0.01). There was no significant difference between the two groups in HRQoL scores (PCS, MCS) at baseline and 3-month assessment (both p > 0.05).

Findings from the regression analyses of factors associated with PCS scores are presented in Table 4. Due to the correlation between GOHAI and OHIP-14, GOHAI and OHIP-14 scores as a factor of HRQoL should be added into the models separately with the other factors. Consequently, two final models were parallel generated. In the final model with baseline OHIP-14 scores as an independent variable, those who were in the group of AOHCP had a PCS score higher than those who were in the group of COHCP by a value of 3.31 (p < 0.05). Every increase in baseline PCS score of 1 was associated with an increase in PCS scores at 3 months by a value of 0.53 (p < 0.001). Every increase in baseline GOHAI score of 1 was associated with an increase in PCS scores at 3 months by a value of 0.34 (p < 0.01). In the final model with baseline OHIP-14 scores as an independent variable, those who received the AOHCP had a PCS score value higher than those who received the COHCP by a value of 3.63 (p < 0.05). Every increase in baseline PCS score by a value of 1 was associated with an increase in PCS scores at 3 months by a value of 0.56 (p < 0.001). Every increase in baseline OHIP-14 score by a value of 1 was associated with a decrease in PCS scores at 3 months by a value of 0.26 (p < 0.05). Similar effects of intervention, baseline GOHAI score, and baseline OHIP-14 score on PCS at 6 months were also observed.

**Table 4:** Factors associated with PCS scores (linear regression model).

Model	Variable	Unstandardized coefficient	SE	Standardized coefficient	p-value	Collinearity statistics	
						Tolerance	VIF
**3 months**							
**A**	Intervention	−3.31	1.47	−0.19	0.027	1.00	1.00
	PCS baseline	0.53	0.10	0.45	<0.001	0.93	1.08
	GOHAI baseline	0.34	0.12	0.24	0.007	0.93	1.07
**B**	Intervention	−3.63	1.49	−0.21	0.017	0.99	1.01
	PCS baseline	0.56	0.10	0.48	<0.001	0.96	1.04
	OHIP baseline	−0.26	0.11	−0.21	0.018	0.96	1.04
**6 months**							
**A**	Intervention	−3.92	1.80	−0.21	0.032	1.00	1.00
	GOHAI baseline	0.39	0.15	0.26	0.011	0.93	1.07
	PCS baseline	0.29	0.13	0.23	0.025	0.93	1.08
**B**	Intervention	−4.35	1.80	−0.23	0.018	0.99	1.01
	OHIP-14 baseline	−0.34	0.13	−0.26	0.010	0.96	1.04
	PCS baseline	0.31	0.12	0.24	0.014	0.96	1.04

3-month final model: adjusted RP Psquare (model 3^a^) = 0.33; adjusted R square (model 3^b^) = 0.31. 6-month final model: adjusted RP Psquare (model 3^a^) = 0.16; adjusted R square (model 3^b^) = 0.16.

Findings from the regression analyses of factors associated with MCS scores are presented in Table 5. Unlike PCS, intervention group was not significantly associated with MCS scores at 3 and 6 months (p > 0.05). In the final model, baseline MCS score and baseline numbers of comorbidities was associated with MCS score at 3 months. Every increase in baseline MCS score by a value of 1 was associated with an increase in MCS scores at 3 months by a value of 0.58 (p < 0.001). Every increase in baseline numbers of comorbidities was associated with a decrease in MCS scores at 3 months by a value of 2.04 (p < 0.05). Similar effects of baseline MCS score and number of comorbidities on MCS at 6 months were also observed.

**Table 5:** Factors associated with MCS scores (linear regression model). 3-month model: adjusted R square = 0.45. 6-month model: adjusted R square = 0.37.

Model	Variable	Unstandardized coefficient	SE	Standardized coefficient	p-value	Collinearity statistics	
						Tolerance	VIF
**3 months**	Intervention	0.02	1.55	0.01	0.99	0.98	1.02
	MCS baseline	0.58	0.07	0.66	<0.001	1	1
	Comorbidities	−2.04	0.84	−0.19	0.017	0.98	1.02
**6 months**	Intervention	0.15	1.68	0.01	0.93	0.97	1.03
	MCS baseline	0.5	0.08	0.56	<0.001	0.96	1.04
	Comorbidities	−3.8	0.93	−0.35	<0.001	0.94	1.06
	Brushing habit	6.17	1.94	0.28	0.002	0.91	1.1

## Discussion

In assessing oral health-related quality of life (OHRQoL), three instruments were employed: GOHAI, OHIP-14, and OHTS. The use of two or more measures of OHRQoL is advocated as it provides a more comprehensive assessment of OHRQoL. Furthermore, given that increasingly OHRQoL is used as an outcome measure of interventions, it is important to decipher whether one specific measure is more sensitive and responsive than the other to the interventions under study.

The baseline median GOHAI score was 54.0 and comparable to the findings among stroke patients at the time of discharge from hospital - a median GOHAI score of 52^9^. The baseline mean GOHAI score was 51.7, and higher than what has been reported during acute phase of stroke - a mean GOHAI score of 45.6^15^, indicating better OHRQoL among patients with stroke during outpatient rehabilitation. Likewise, the baseline median OHIP-14 was 4.0, lower than what has been reported among hospitalized stroke patients - a median OHIP-14 score of 7.0^13^, again indicating better OHRQoL among patients with stroke receiving outpatient rehabilitation. Both GOHAI and OHIP-14 scores can be interpreted as a low level of OHRQoL impairment among study participants at baseline. OHTS assessment suggested substantial OHRQoL impairment compared to findings of GOHAI and OHIP-14 assessments as approximately a third of subjects perceived that their oral health was worse following stroke at baseline. This is likely to be attributed to that OHTS assessed perceived change or transition following stroke rather than perceived OHRQoL at specific time point with no reference to the stroke event^9^.

Findings from the regression analyses identified a significant association between intervention and OHRQoL at the end of the clinical trial when assessed by OHTS but not by GOHAI or OHIP-14. This suggests that OHTS is a more sensitive to oral hygiene care intervention than GOHAI or OHIP-14. Also, OHIP-14 and GOHAI baseline scores among participants in this study were comparable to ‘healthy’ Chinese elderly in Hong Kong and China^16–18^. The ‘high floor effect’ (no perceived negative impact) hampers their ability to capture the improvements caused by interventions. At the end of the observation period, findings from the regression analyses identified that a significant association between intervention and OHRQoL at 6 months in terms of GOHAI but not in terms of OHTS and OHIP-14. Interestingly a significant association was observed between intervention and GOHAI scores at 6 months while significant association between the intervention group and GOHAI scores at 3 months was not evident. A suggested explanation of this is a latent effect of the intervention on OHRQoL as assessed by GOHAI. No significant association was observed between intervention and OHIP-14 scores at 6 months. A possible reason is that GOHAI assesses largely physical aspects whereas OHIP-14 focuses on psychological and social aspects, and thus is less sensitive to the subtle changes that oral hygiene care may produce in psychosocial aspects.

Some other factors were also significantly associated with OHQoL both at the end of the clinical trial period and at the end of the observational period. Baseline OHRQoL values was associated with OHRQoL at the end of the clinical trial and at the end of the observational period, irrespective of how OHRQoL was assessed. This is not surprising as baseline value is predictive of future states. Baseline plaque levels were associated with subsequent GOHAI values at 3 and 6 months suggesting that GOHAI may be more sensitive to underlying clinical state than the other measures. Numbers of comorbidities at baseline was associated with OHTS scores at 6 months. This highlights the potential effect of systemic diseases on OHRQoL as were reported by other studies^19–21^. Clinical oral health indicators, such as DMFT, CPI, and LOA, were not significantly associated with OHRQoL scores both at the end of the clinical trial period and the observational period. This concurs with findings in previous studies in the UK, Greece, and Thailand, which suggest that OHRQoL may not be sensitive to some clinical oral health indicators of elderly^22–24^.

When interpreting the findings of this randomized controlled trial (RCT) in light of other RCTs relating to OHRQoL, some comparisons can be made. Firstly, examining the effect of COHCP (manual toothbrushing), the finding was that it was not effective in improving OHRQoL (in terms in differences in GOHAI, OHIP-14 and OHTS scores). There are no comparable studies in the literature to support or refute this finding. Secondly, AOHCP (powered toothbrushing and chlorhexidine mouth rinsing) was effective in improving OHRQoL (in terms in changes in GOHAI, OHIP-14 and OHTS scores) as demonstrated by within group comparison. This concurs with the findings of Lam, *et al*.^13^ who reported significant improvements in OHRQoL (as assessed by OHIP-14) following the use of powered toothbrush and chlorhexidine mouthrinse. Findings from the regression analyses (controlling for other factors) provides further evidence to support the use of AOHCP over COHCP on the basis that there was significantly higher OHRQoL (OHTS) at the end of the clinical trial period. Moreover that AOHCP appeared to have a lasting effect on OHRQoL in terms of higher GOHAI scores at the 6-month assessment. Of note, while a statistical significant association between intervention and OHRQoL in terms of OHTS and GOHAI was observed, the magnitude of difference in scores was relatively small and needs further investigation as to whether such a difference could be interpreted to be clinically important^25^.

Among participants of this study, their baseline PCS scores ranged from 19.0 to 53.0, with a median value of 33.5 and mean value of 33.7. This is indicative of poorer physical health with reference to the mean PCS value of 50.2 for the general population in Hong Kong^26^. Moreover, the mean PCS value was lower than findings among Hong Kong people with pulmonary diseases (43.9), joint disease (42.6) or other chronic disease (44.7). The PCS values are comparable to other stroke population among non-institutionalized stroke survivors in the US with a mean PCS value of 35.6^27^. However, lower PCS values (mean 29.4, median 28.2) have been reported among hospitalized stroke patients in Hong Kong^13, 15^. The median MCS was 44.3 among all participants at baseline. This is indicative of poorer mental health with reference to the mean MCS value of 48.4 for general population in Hong Kong^26^. Moreover, MCS values were lower than findings among Hong Kong people with pulmonary diseases (46.3), joint disease (49.0) or other chronic disease (49.1) but obviously higher than those with psychological diseases (42.7). The MCS values are comparable to non-institutionalized US stroke survivors^27^, but lower than findings among community-dwelling US stroke survivors^28^.

There were significant improvements in PCS and MCS score among all participants. This concurs with other reports of improvement in physical health and mental health following rehabilitation among stroke survivors^29, 30^. Of note an observed greater significant magnitude of improvement was in physical health than mental health. This may be due to the physical exercise components of the general rehabilitation programmes, although it is acknowledged that there are some mental health benefits from such physical exercises as well.

Regression analyses found that intervention was significantly associated with PCS values at 3 and 6 months accounting for other factors in the model. Participants in the group of AOHCP had a higher PCS by a value of ~4 than participants of the COHCP group both at 3 months and 6 months. This is worthy of further investigation to decipher pathways of association (direct or indirect associations) in PATH analyses as to whether AOHCP had direct effect or indirect effect via OHRQoL on HRQoL. As expected baseline PCS and OHRQoL values (baseline OHIP-14 and GOHAI scores) were significantly associated with PCS values at 3 and 6 months. Regression analyses did not identify association between intervention and MCS values at the end of the clinical trial or the observational period. It implies that the intervention may not be able to address the mental component of general health. Numbers of comorbidities at baseline was associated with MCS values at 3 months and at 6 months. Increase in numbers of comorbidities by a value of 1 was associated with a change in MCS by a value of ~2 at 3 months and ~4 at 6 months. This concurs with findings of OHRQoL as assessed by OHTS. It highlights additional challenges of recovery from stroke with existing comorbidities as reported by other studies^31, 32^.

The sample size of this study was calculated to detect a significant difference in the primary outcome variable - the level of dental plaque between the two groups at three-month review. The part regarding the outcome of clinical oral health (e.g. level of dental plaque, gingival bleeding, DMFT, CPI and *etc*.) was published previously^33^. Therefore, the sample size may be relatively small to detect significant difference in the outcomes of oral health- and health-related quality of life, and the finding may be preliminary. Future studies with sample size based on primary outcome of oral health- and health-related quality may be needed.

## Methods

The study gained ethical approval from *the Institutional Review Board (IRB) of the University of Hong Kong* (IRB reference number: UW 12–090). This clinical trial was conducted according to the guidelines and regulation of *the Institutional Review Board of the University of Hong Kong*. This trial was registered both at the *Hong Kong Clinical Trial Register* (Registration No.: 003900, Registration Date: Feb 20, 2014) and *ClinicalTrials.gov* (Registration No.: NCT03003871, Registration Date: September 9, 2016). The full protocol can be accessed through *ClinicalTrials.gov Protocol Registration and Results System (PRS)*. In brief, this study was a 3-month parallel single blinded randomized controlled clinical trial, followed by a 3-month observational study. The study was conducted at the *Mrs Ng Memorial Day Outpatients Center*, *Tung Wah Hospital* (TWH) in Hong Kong SAR. Patients with stroke who are discharged but have sustained functional impairments receive further outpatient rehabilitation at this centre. Subjects were recruited according to the following inclusion/exclusion criteria: 1. admitted in the outpatient stroke rehabilitation programme within 6 months; 2. *Barthel Index* (BI) scores of <70; 3. having at least one tooth; 4. *Mini Mental State Examination* (MMSE) >18; 5. being able to follow a one-step command (as an assessment of communication); 6. no indwelling naso-gastric feeding tubes. Patients were recruited and screened by an experienced dental surgery assistant in the study team. Subjects who satisfy the criteria were invited to participate in this study by an experienced dental surgery assistant. They were informed of potential benefits and adverse effects of the interventions, the procedures, and the anticipated outcomes of this study. If subjects agreed to be enrolled in this study, they were asked to provide their written informed consent.

Sample size calculation was calculated based on the levels of plaque at 3 months. Participants were randomly assigned to one of the two group: (1) a conventional oral hygiene care programme (COHCP) - supply of a manual toothbrush (Oral-B^®^ Pro-Health All-In-One), a standardized toothpaste (Colgate^®^ Maximum Cavity Protection), and oral hygiene training, or (2) an advanced oral hygiene care programmes (AOHCP) - supply of a powered toothbrush (Oral-B^®^ AdvancePower^TM^ 400 series), 0.2% *chlorhexidine gluconate* mouth rinse (Corsodyl^®^), a standardized toothpaste (Colgate^®^ Maximum Cavity Protection) and oral hygiene training. Block randomization with the block size of 4 (ABBA) was adopted. Oral hygiene training was offered by an experienced dental surgery assistance, who was not involved in the questionnaire interview. Toothbrushing practice was firstly demonstrated on tooth block models. Each subject was asked to demonstrate their toothbrushing skills on tooth block models. For those assigned to AOHCP, they received specific manufacturer’s instructions on powered toothbrush. In addition, they were instructed to rinse twice daily with 10 ml chlorhexidine (at least 30 minutes after brushing).

The randomized sequence was computer generated by the principle investigator. The number of allocation sequence was sealed in an opaque envelope. A nurse at the rehabilitation centre who was independent of the research team was responsible for dispensing the allocation number to subjects. After randomization and baseline assessment, intervention commenced and lasted for three months. After 3 months, observational period started and no study articles were provided to subjects. Follow-up reviews were schedule at 3 months and 6 months after the commencement of intervention.

The subjective health assessment included HRQL and OHRQL assessments. This consisted of the Cantonese versions of the Short Form Health Survey 12: SF-12^26^, the Oral Health Impact Profile 14: OHIP-14^16^, the Geriatric Oral Health Assessment Index: GOHAI^17^, and Oral Health Transition Scale (OHTS)^9^. The questionnaires were interviewed by a researcher who was unaware of the patients’ treatment assignment (single blind).

The SF-12 consists of 12 items covering eight conceptual domains of health: general health (GH), physical functioning (PF), bodily pain (BP), role-physical (RP), mental health (MH), vitality (VT), social functioning (SF), and role-emotional (RE). Four items are reversed for scoring (item no.: 1, 8, 9, and 10). Each item has its own physical component summary (PCS) and mental component summary (MCS) regression coefficients^34^. The response to each item was multiplied by its PCS regression coefficient, and added together with a PCS constant value to provide physical component summary scores (PCS)^35^. Likewise, each item was multiplied by its MCS regression coefficient, and added together with a MCS constant value to provide Mental Health summary scores (MCS). A higher sum scores represents better HRQoL.

The GOHAI comprising of 12 items assessed three dimensions of the oral health impact: physical functions, psychosocial functions, and pain or discomfort. Responses to the frequency of an event occurring as described by the item are coded using a 5-point Likert scale: 1 = always, 2 = often, 3 = sometimes, 4 = seldom, and 5 = never. The responses to nine items (limit food due to dental problems, trouble biting and chewing, used medication, sensitive to temperature, nervous due to teeth, uncomfortable eating with people, prevented from speaking, worried about teeth, and limited contacts with people) should be scored reversely. Summary GOHAI scores were also derived by summating responses to items after reversing the coding of the nine items. A higher sum scores represents better OHRQoL. The OHIP-14 assesses seven dimensions of oral health impairment^36^: functional limitation, physical pain, psychological discomfort, physical disability, psychological disability, social disability, and handicap. Responses to the frequency of an event occurring as described by the item are coded on a 5-point Likert scale: 0 = never, 1 = hardly ever, 2 = occasionally, 3 = fairly often, and 4 = very often/all of the time. Summary OHIP-14 score were derived by summating responses to each item. A lower sum score represents better OHRQoL. The OHTS comprises eight questions which evaluate the patient’s perceptions before and after the stroke regarding their general appearance, general oral health, and general comfort of their mouth, ability to chew hard and soft foods, speak, swallow, and use a toothbrush. Responses to each item are coded on a 5-point Likert scale: 1 = much better, 2 = somewhat better, 3 = no difference, 4 = somewhat worse, 5 = much worse. Summary OHTS scores were derived by adding responses to each item. Similarly, a lower sum score represents better OHRQoL.

In this study, the primary outcome is OHQoL and HQoL at 3 months, and the secondary outcome is at OHQoL and HQoL 6 months. In bivariate analyses, within and between groups comparisons were made. Wilcoxon Signed Rank test for related samples and Mann Whitney U test for independent samples were performed since all the dependent variables in the binary analyses were continuous but did not follow a normal distribution. Linear regression was adopted when the dependent variables were continuous and residuals of the regression followed a normal distribution. Negative binomial regression was adopted when the continuous dependent variables had over-dispersed count data. Missing outcomes imputation at follow-up reviews in regression analysis used *Last Observation Carried Forward* (LOCF) method. The model fitting adopted a forward-wald method. The factor of intervention, which is of our core interest, is fixed in the model and one of the rest explanatory factors added into the model each time. The coefficient of the independent variables with the smallest significant p value (significance level p < 0.05) was entered into the regression model. The model fitting process was repeated again until no more independent variable had significant p values. The statistics software packages SPSS 21 for Windows (SPSS Inc., Chicago, USA) was employed. The datasets generated analysed in this study are available from the corresponding author on reasonable request.
